# Intracranial myxoid mesenchymal neoplasms with *EWSR1* gene rearrangement: report of 2 midline cases with one demonstrating durable response to MET inhibitor monotherapy

**DOI:** 10.1093/noajnl/vdab016

**Published:** 2021-02-04

**Authors:** Yanel De Los Santos, David Shin, Samuel Malnik, Marie Rivera-Zengotita, David Tran, Ashley Ghiaseddin, Jesse Lee Kresak

**Affiliations:** 1 Department of Pathology, University of Florida, Gainesville, Florida, USA; 2 Department of Neurosurgery, University of Florida, Gainesville, Florida, USA; 3 College of Medicine, University of Florida, Gainesville, Florida, USA

**Keywords:** EWSR rearrangement, intracranial myxoid mesenchymal neoplasms, MET inhibitor monotherapy

EWSR1 fusions have been identified in multiple neoplasms involving bone and soft tissue. Recently, cases of intracranial myxoid mesenchymal neoplasms harboring *EWSR1-CREB* family gene fusions have been reported in young patients with histologic features reminiscent of the myxoid variant of angiomatoid fibrous histiocytoma. Here, we report 2 cases of adult males with midline intracranial EWSR1 myxoid neoplasms. Patient 1 (age 37) presented with headaches and MRI demonstrated an enhancing right thalamic mass extending into the quadrigeminal cistern. Near-total resection was achieved, and residual disease was treated with stereotactic radiosurgery (SRS). Five-year follow-up is negative for recurrence. Patient 2 (age 28) had a past medical history significant for high-grade B-cell lymphoma of his femur and kidney for which he received systemic and intrathecal treatment. He presented to our institution with headaches, visual changes, nausea, and vomiting, and MRI demonstrated a homogenously enhancing intraventricular tumor. Gross total resection was achieved, yet despite postoperative radiation therapy, he progressed 11 months later. He was then treated with radiosurgery and crizotinib, leading to stable disease 23 months after start of targeted therapy. In both cases, *EWSR1* gene rearrangement was identified by fluorescence in-situ hybridization (FISH). Reverse transcription polymerase chain reaction (rt-PCR) showed a CREB1 gene fusion partner in both cases. Our cases expand the clinicopathologic features of these newly recognized tumors and include an older age at presentation and a relatively elevated proliferation index. We also present a durable response to monotherapy with a MET inhibitor in response to this gene fusion.

EWSR1-CREB1 family gene fusions have recently been identified in myxoid mesenchymal neoplasms in the CNS. Gene fusions between the Ewing sarcoma breakpoint region 1 gene (EWSR1) and members of the cAMP response element binding (CREB) family, which include CREB1, CREM, and ATF1, are not unique to this neoplasm as they have been identified in multiple other tumors including clear cell sarcoma of the gastrointestinal tract, hyalinizing clear cell sarcoma of the salivary gland, primary pulmonary myxoid sarcoma, and angiomatoid fibrous histiocytoma. These neoplasms have different clinical presentations, histologic morphology, and patient outcomes, despite sharing a common molecular alteration. Likewise, EWSR1-rearranged myxoid mesenchymal neoplasms of the CNS have distinct histologic features and have a reported propensity for the supratentorial region of adolescents and young adults. The upcoming World Health Organization (WHO) classification of tumors for CNS may have a different nomenclature for these tumors, possibly angiomatoid fibrous histiocytoma/intracranial myxoid mesenchymal tumor. Here, we present 2 cases of intracranial myxoid mesenchymal tumor with EWSR1-CREB1 fusions that expand the clinical presentation and provide therapeutic outcomes.

## Case Report 1

**Figure 1.**
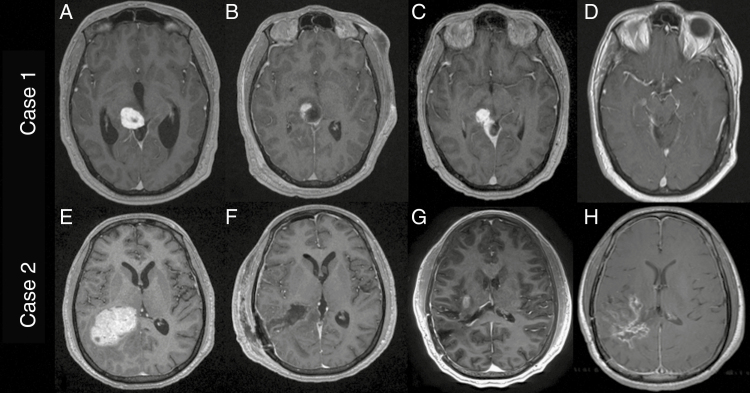
Case 1: T1 post-contrast MRI images of case 1 demonstrating initial tumor (a), immediate post op (b), recurrence at 3 months (c), and 52 months after SRS showing no progression (d). Case 2: T1 post-contrast MRI images of case 2 demonstrating pre-op (a), post-op (b), recurrence at 11 months (c), and stable disease burden on crizotinib monotherapy at 23 (d)

A 37-year-old male presented with a 2-month history of new onset migraines, neck pain, and right eye pressure. As a part of his ophthalmologic work-up, he underwent brain MRI demonstrating a 1.7 × 1.6 × 1.5 cm well-circumscribed homogenously enhancing mass centered in the pulvinar of the right thalamus extending into the adjacent quadrigeminal cistern and abutting the pineal gland with associated ventriculomegaly (Figure 1a). He underwent endoscopic third ventriculostomy and stereotactic biopsy and proceeded to have an unremarkable hospital course. He was discharged the following day.

**Figure 2.**
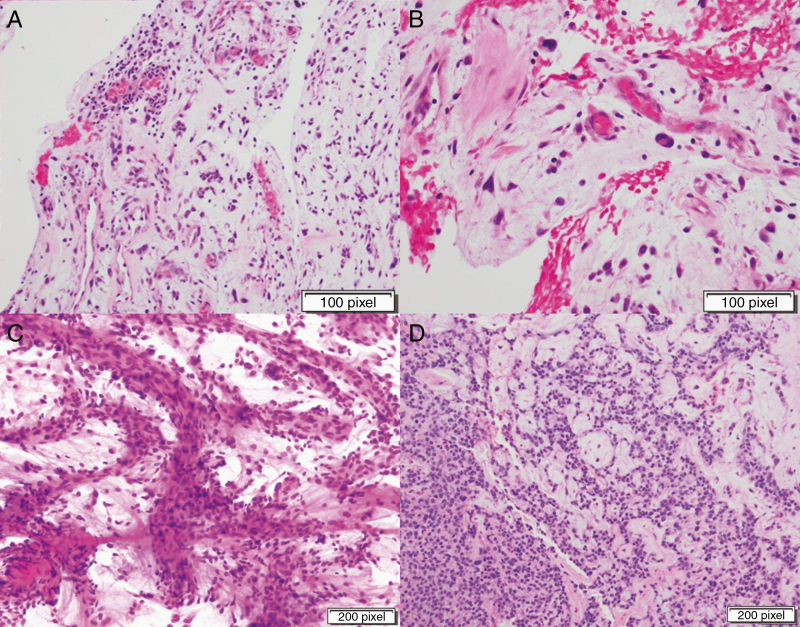
Case 1: (a,b). Hematoxylin and Eosin (H&E) photomicrographs of the lesion in case 1 showing small atypical, focally cohesive spindled and stellate cells embedded within a myxoid matrix with focal microvascular proliferation and non-specific perivascular inflammation. Case 2: (c,d) Squash preparation and H&E photomicrographs of the lesion in case 2 showing nests and cords of epithelioid cells with scant eosinophilic cytoplasm and abundant myxoid stroma. Prominent small and medium-sized blood vessels were seen and in areas, tumor cells also displayed sheet-like/solid growth pattern. Mitoses were easily identified on routine H&E stains (up to 5 mitoses per 10 high power fields).

Pathology of the lesion demonstrated small atypical, focally cohesive spindled and stellate cells embedded within a myxoid matrix with focal microvascular proliferation and non-specific perivascular inflammation. Few mitoses were identified within the atypical cells and endothelial cells (Figure 2a,b). The Ki-67 proliferation index was approximately 6%. Immunohistochemical stain for EMA were weakly positive in scattered cells, and immunostains for GFAP, S100, CD34, ERG, PR, synaptophysin, AE1/3, CAM 5.2, SMA, and Sall-4 were negative.

After discussion of his case at our institution’s tumor board, surgical resection was recommended based on the pathologic findings. He then underwent a right retrosigmoid craniotomy for the lesion. He received dexamethasone and hyperosmolar therapy to treat his edema and underwent MRI demonstrating near-total resection (Figure 1b). He had a prolonged postoperative course requiring tracheostomy and gastrostomy placement during his hospitalization. Pathology on the resection specimen was similar to the biopsy. Fluorescence in-situ hybridization was performed on the resected lesion revealing a positive EWSR1 rearrangement (chromosome 22q12.2) and reverse transcription polymerase chain reaction (rt-PCR) confirmed EWSR1-CREB1 gene fusion.

At his 3-month follow-up, he had improved neurologically to the point where his tracheostomy and gastrostomy had been removed. Unfortunately, MRI revealed progression of his residual disease (Figure 1c) which was then treated with SRS for a total of 12.5 Gy. He has since had multiple interval images which show no evidence of disease progression at 52 months (Figure 1d).

## Case Report 2

A 28-year-old male with a history of high-grade Burkitt lymphoma of his femur and kidney, for which he received intrathecal methotrexate and systemic vincristine and cyclophosphamide ten years prior, presented to an outside hospital with new onset headaches, associated vision changes, nausea, vomiting, and at least one documented seizure episode. MRI demonstrated an avidly enhancing, lobulated, intraventricular mass measuring 5.7 × 4.8 × 2.7 cm (Figure 1e) centered in the right atrium. Complete neuraxial imaging was negative for any other lesions. He subsequently underwent right parietooccipital craniotomy with gross total resection (Figure 1f). Postoperatively he was found to have a left homonymous hemianopsia but no other discernable neurologic deficits. Eventually, he was discharged from the hospital to home on post-op day 8.

The final pathology demonstrated a neoplasm composed of epithelioid cells with scant eosinophilic cytoplasm and abundant myxoid stroma arranged in nests, cords, and focally sheet-like growth patterns. Prominent small- and medium-sized blood vessels were seen. Mitoses were readily identified on routine hematoxylin and eosin (H&E) stains (up to 5 mitoses per 10 high power fields, Figure 2c,d). Tumor cells were strongly immunoreactive for vimentin and focally positive for EMA. Additionally, the tumor was negative for GFAP, S-100, CAM5.2, brachyury, PR, transthyretin, synaptophysin, pan-cytokeratin, BRAFV600E mutation, and ALK. Ki-67 demonstrated an elevated proliferation index of 5%–10%. Molecular testing was performed on this tumor (Foundation One) and showed no reportable genomic alterations. Reverse transcription polymerase chain reaction (rt-PCR) demonstrated an EWSR1-CREB1 gene fusion.

Upon evaluation at his 6-week follow-up, his visual field deficit had completely resolved. At the recommendation of our neuro-oncology team, he was referred for adjuvant fractionated radiation treatment and received a total dose of 60 Gy to the resection cavity. This was followed by a period of surveillance without treatment, during which he was clinically stable. However, at 11 months postoperatively, his routine imaging revealed disease progression (Figure 1f). He then underwent SRS in a single 20 Gy dose.

However, in the months following his SRS, he began to develop worsening left hemiparesis. Imaging revealed continued growth of his right sided lesion as well as an increase in surrounding edema. Having no surgical options, the decision was made to start crizotinib based on his EWSR gene rearrangement. Bevacizumab was briefly used due to worsening symptomatic vasogenic edema. Ultimately, he continued on crizotinib monotherapy, and his hemiparesis gradually improved. Twenty-three months since starting crizotinib, the patient remains clinically and radiographically stable (Figure 1g).

## Discussion

Intracranial myxoid mesenchymal tumor with EWSR1 fusion is a relatively new entity first described in 2017.^1^ The entity has caused some controversy due to its common molecular alteration and similar histomorphology as the myxoid variant of angiomatoid fibrous histiocytoma (AFH).^2^ In contrast to AFH, intracranial EWSR1 myxoid mesenchymal tumors tend to lack lymphocytic cuffing and a prominent vascular component histologically. Also, in the initial series of myxoid AFH, none of the tumors were located intracranially.^3^ In addition, myxoid AFH is reported to have rare local recurrences, whereas 25% (3 of the now 12) reported intracranial neoplasm have recurrences.^4,5^ And, as our repertoire of molecularly defined tumor types expands, we know that the shared gene fusion, such as that identified on our tumor type, AFH, CCS, GICSS, and PPMS, does not equate to a shared tumor family.

Fifteen cases of intracranial myxoid mesenchymal tumors have been reported. The age range in the literature is 12–67 years, and the majority are supratentorial. With the addition of our 2 cases, this neoplasm demonstrates an overall 1:1 predominance. All reported cases are multinodular, lack lymphocyte cuffing, and have myxoid backgrounds. Only 5 reported cases have an increased proliferative index, yet none display necrosis. A majority of reported cases lack pseudoangiomatoid spaces, a pseudocapsule, and have a collagen component. Most reported cases have EMA and desmin positivity and EWSR1 fusion partners include CREB1, CREM, and ATF1 with CREB1 being the most common (Table 1).^1–11^

**Table 1.** Published Cases of Intracranial Myxoid Mesenchymal Neoplasms

Author	Age	Sex	Location	Mitoses	EMA	Desmin	EWSR1-CREB	Long-Term Recurrence
Kao et al.	15	F	Dural-based	−	+	+	EWSR1-CREM	N/A
	23	F	Dural-based	−	+	+	EWSR1-CREB1	N/A
	20	M	Frontal	−	+	+	EWSR1-CREB1	N/A
	12	M	Frontal	−	+	−	EWSR1-ATF1	N/A
Bale et al. (8)	12	M	Dural-based	+	+	+	ESWR1-CREB1	N/A
	14	F	Ventricular	−	+	+	ESWR1-CREB1	N/A
	18	M	Frontal	−	+	+	ESWR1-CREM	N/A
Sciot et al.	17	F	Frontal	−	+	−	EWSR1-ATF1	+
Gareton et al.	19	M	Dural (CPA)	+	+	−	EWSR1-CREM	+
White et al.	9	M	Frontal	−	+	+	EWSR1-CREM	N/A
Ballester et al.	67	M	Dural-based	+	+	+	EWSR1-ATF1	N/A
Komatsu et al.	53	F	Ventricular	−	+	+	EWSR1-CREB1	N/A
Valente et al.	58	F	Ventricular	+	+	+	EWSR1-CREB1	N/A
Ward et al.	48	F	Ventricular	+	−	−	EWSR1-ATF1	+
Libbrecht et al.	14	F	Dural-based	−	+	+	EWSR1-CREB1	N/A
Patient 1	37	M	Thalamic	+	+	N/A	EWSR1-CREB1	−
Patient 2	28	M	Ventricular	+	+	N/A	ESWR1-CREB1	+

Our cases have similar characteristics to the previously described cases such as shared histopathology, an intracranial location, and having EWSR1-CREB1 gene fusion. Both of our cases were midline tumors, and both our cases occurred in slightly older adults than most reported cases. Our cases also differ from those previously reported cases in that they have somewhat higher proliferative indices and mitotic activity. Interestingly, case 2 occurred in a patient with a remote history of Burkitt lymphoma treated with intrathecal and systemic chemotherapy. This association to childhood malignancy was also reported in another case with the development of an intracranial EWSR1 tumor 5 years after treatment for neuroblastoma.^2^ Lastly, our cases demonstrate successful disease control following surgical resection and radiotherapy in one patient, as well as stable disease in a patient who received crizotinib monotherapy, also after surgical resection and radiotherapy. Crizotinib is a tyrosine kinase receptor inhibitor with multitargeted action at ALK (anaplastic lymphoma kinase), c-MET (mesenchymal epithelial transition growth factor), and ROS1 (c-ros oncogene 1). Indications for the drug are based on clinical trials for ALK-positive non-small cell lung cancer (NSCLC). However, case reports have discussed responses with crizotinib in ALK-positive anaplastic large cell lymphoma (ALCL) as well as ALK-translocated inflammatory myofibroblastic tumor (IMT). There are clinical trials being performed with Crizotinib in neuroblastoma given the suspected role ALK mutations play in the malignancy of this disease.^12^ Of interest to our case report, recent work was published highlighting ALK mutations being over expressed in angiomatoid fibrous histiocytoma (AFH) raising questions of therapeutic potential. Since EWSR1 rearrangement is present in majority of AFH cases, further research is needed to determine the relationship between ALK and EWSR1 fusion proteins and whether the former may act as downstream target for the latter.^13^ There is also a report of using crizotinib to achieve long-term durable response in EWSR1-CREB1 fusion in a patient with gastrointestinal neuroectodermal tumor.^14^ The presumed mechanism is that this fusion product results in upregulation of the MET pathway, sensitizing it to a receptor tyrosine kinase inhibitor such as crizotinib. Our goal for precision medicine treatment with crizotinib was 24 months but discontinued after 23 months because the patient had been experiencing fatigue, a known adverse effect with this drug.^12^ Although the patient was still working, there was a desire on the patient’s part to return to more normalcy after completing nearly 2 years of treatment. Ultimately, decision was made to continue off treatment with surveillance monitoring. Nonetheless, this second case highlights a durable response to personalized and targeted treatment paradigm and lends credence to the hypothesis that MET inhibitors can be leveraged effectively in the setting of EWSR1-CREB1 fusion.

## Conclusion

We present 2 cases of intracranial myxoid mesenchymal tumor with EWSR1-CREB gene fusion who have long-term follow-up currently demonstrating an absence of progression in both, one of which in response to targeted crizotinib monotherapy.



**Conflict of interest statement.** D.T. has received grant funding from Celldex, NWBiotech, Novocure, and Merck; he has received personal fees from Novocure and prIME Oncology. A.G. has received personal fees from Novocure and Monteris Medical. He has also received research funding support from Orbus Therapeutics. The other authors declared no conflict of interest.
